# Supplementary material for: Survivin Overexpression Has a Negative Effect on Feline Calicivirus Infection
Source: Viruses. 2019 Oct 30;11(11):996. doi: 10.3390/v11110996 (PMC6893618; doi:10.3390/v11110996)
Supplement: Supplementary file 1 [file viruses-11-00996-s001.pdf]

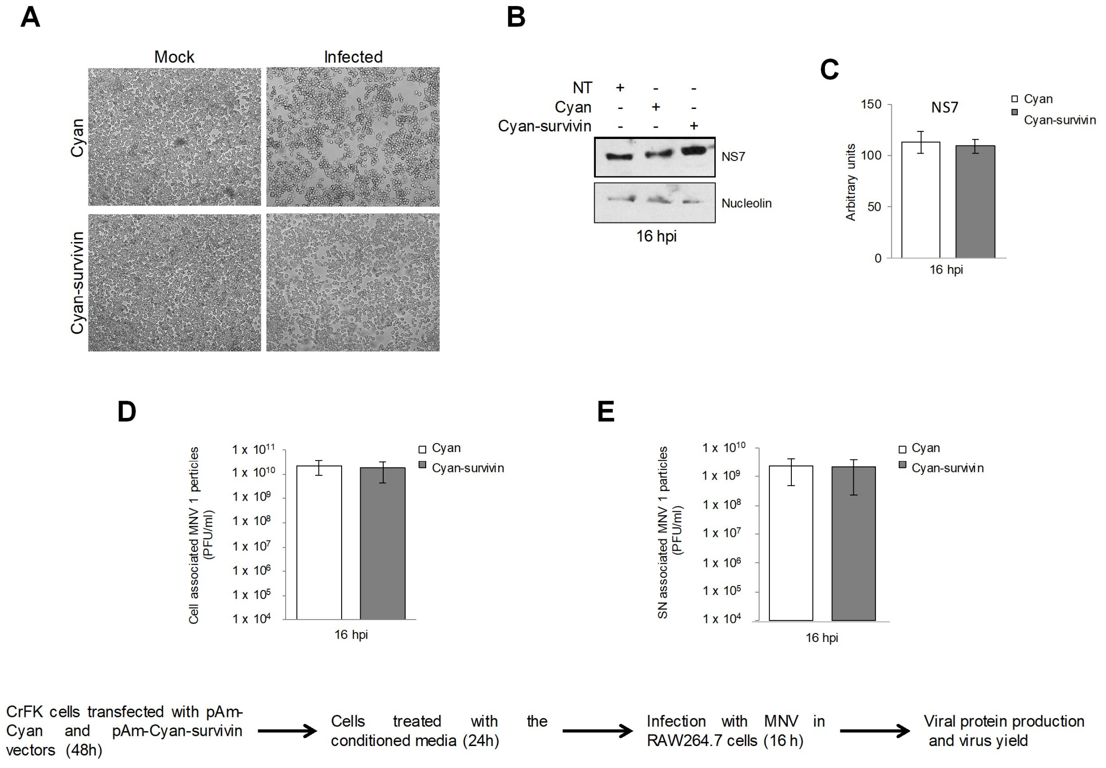

Supplementary Figure 1. MNV viral protein and viral yield is not altered in RAW 264.7 cells treated with supernatants form CrFK cells overexpressing survivin. RAW 264.7 cells were treated with supernatants form CrFK cells transfected with pAm-Cyan (white) and pAm-Cyan-survivin vectors (grey) for 48 h, and infected with MNV-1 at an MOI of 5 for 16 h. (**A**) Cytophatic effect. (**B**) Total protein extracts were subjected to SDS-PAGE and the levels of NS7 protein were analyzed by western blotting using specific antibodies. (**C**) Band intensities of the scanned images were quantified using ImageJ software and expressed as arbitrary units. Nucleolin was used as the loading control. MNV-1 particles production in (**D**) cell-associated and (**E**) supernatant-associated fractions were quantified by plaque assay. Standard deviations were obtained from duplicates of at least 3 independent assays.

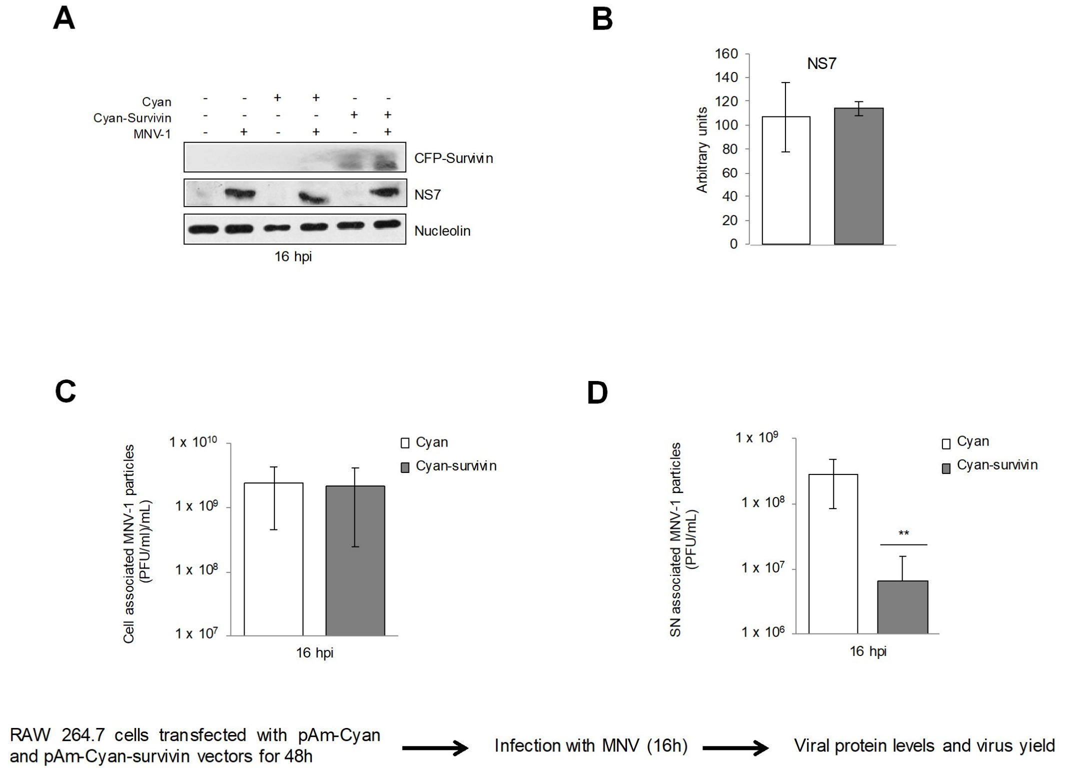

Supplementary Figure 2. The overexpression of survivin does not affect MNV-1 protein or viral production but affects virus release. RAW 264.7 cells were transfected with pAm-Cyan and pAm-Cyan-survivin vectors for 48 h and infected with MNV-1 at an MOI of 5 for 16 h. (**A**) Total protein extracts were subjected to SDS-PAGE and the levels of NS7 protein were analyzed by western blotting using specific antibodies. (**B**) Band intensities of the scanned images were quantified using ImageJ software and expressed as arbitrary units. Nucleolin was used as the loading control. MNV-1 particles production in (**C**) cell-associated and (**D**) supernatant-associated fractions were quantified by plaque assay. ** $P \leq 0.001$ calculated by two way ANOVA. Error bars represent the standard deviation from 3 independent assays.

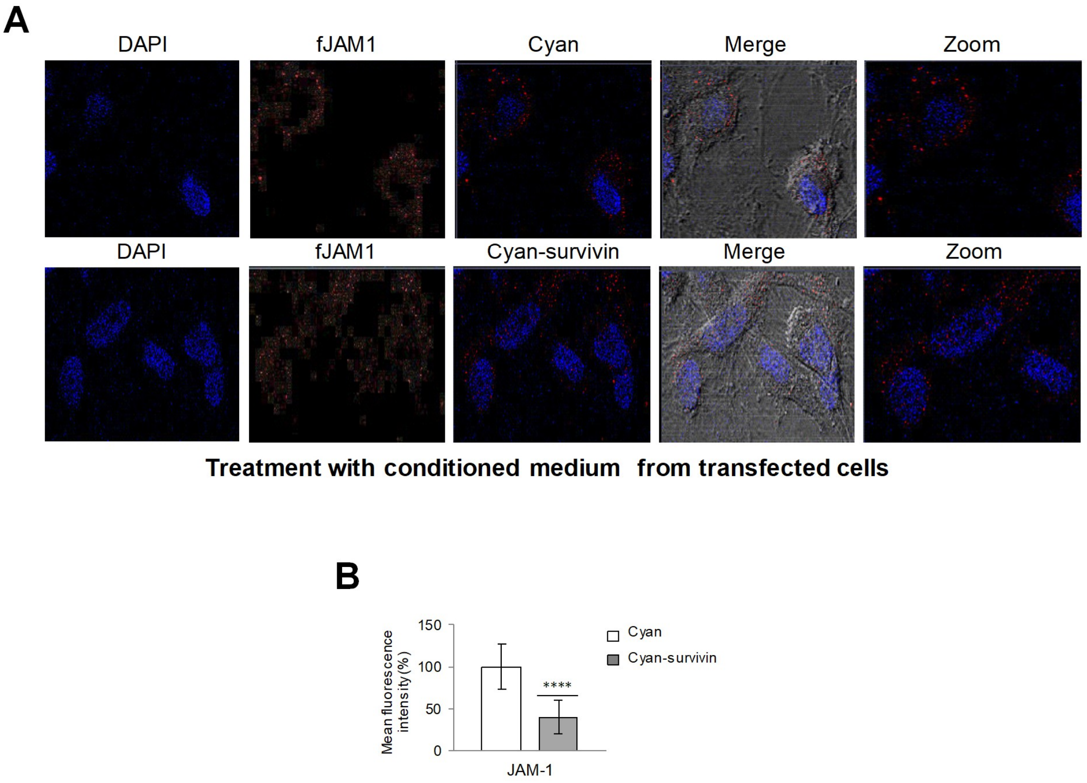

Supplementary Figure 3. The amount of fJAM-1 protein is reduced in CrFK cells treated with conditioned medium from cells overexpressing survivin. (**A**) Non-permeabilized CrFK cells were treated with the conditioned medium from cells transfected with pAm-Cyan and pAm-Cyan-survivin vectors for 24 h and the fJAM-1 molecule in the cell surface was immunostained with an anti-JAM-1 antibody, followed by Alexa Fluor 594 staining (red). DAPI was used to stain nuclei (blue). The cells were analyzed using a Zeiss LSM 700 confocal microscope. The images depict single confocal slices taken from z-stacks. The data shown are representative of at least 3 independent experiments. (**B**) fJAM-1 mean fluorescence intensity was determined by Icy software. The statistical test was performed using the GraphPad Prism 7.00 software. **** $P \leq 0.0001$ calculated by two-way ANOVA. Error bars represent the standard deviation from 3 independent assays.
